# “It made me more confident that I have it under control”: Patient and provider perspectives on moving to a two-drug ART regimen in the United States and Spain

**DOI:** 10.1371/journal.pone.0232473

**Published:** 2020-05-01

**Authors:** Wendy Davis, Andrea Mantsios, Tahilin Karver, Miranda Murray, Yogesh Punekar, Douglas Ward, U. Fritz Bredeek, Santiago Moreno, Dolores Merino, Hernando Knobel, Antonio Campis, Deanna Kerrigan

**Affiliations:** 1 Center on Health, Risk and Society, American University, Washington, D.C., United States of America; 2 ViiV Healthcare, Raleigh, North Carolina, United States of America; 3 Dupont Circle Physicians Group, Washington, D.C., United States of America; 4 Metropolis Medical Group, San Francisco, California, United States of America; 5 Hospital Ramon y Cajal, Madrid, Spain; 6 Hospital Infanta Elena, Huelva, Spain; 7 Hospital del Mar, Barcelona, Spain; 8 Hospital Son Espases, Palma de Mallorca, Spain; Centre for Sexual Health & HIV/AIDS Research, ZIMBABWE

## Abstract

**Background:**

Two-drug regimens (2DR) to treat HIV infection have the potential to reduce long-term toxicity and increase therapeutic options for people living with HIV (PLHIV). Prior phase III trials, SWORD-1 and SWORD-2, as well as GEMINI-1 and GEMINI-2, have demonstrated that a dolutegravir-based 2DR is as effective as three- or four-drug regimens among virologically suppressed patients. Limited information exists, however, on patient and provider experiences with 2DR to inform roll-out and integration into routine clinical care.

**Methods:**

We conducted 39 in-depth interviews with PLHIV currently on 2DR in the context of routine care and 8 of their clinical care providers in the United States (U.S.) and Spain. Participants included 33 male and 6 female PLHIV and 8 providers. Interview topics explored perceptions of and experiences with 2DR compared to prior anti-retroviral regimens (ARVs), side effects, patient satisfaction, and clinical performance. Interviews were audio-recorded, transcribed and analyzed using thematic content analysis.

**Results:**

Participants viewed 2DR as a significant and positive advance, in terms of its ability to effectively treat HIV with reduced toxicity and essentially no reported side effects. Patients noted the central role providers played in the decision to switch to a 2DR regimen and, among U.S. participants, the importance of insurance coverage making this preferred option feasible. Patients and providers agreed that a 2DR regimen would be appropriate for any PLHIV regardless of whether they were treatment naïve or had significant experience with ARVs.

**Conclusions:**

Participants’ experiences with a 2DR regimen were positive with no participants, reporting side effects and all reporting continued viral suppression. Providers valued the reduced toxicity offered by 2DR and served as the primary gateway to a transition to 2DR for patients in both settings. This study provides a foundation for further research on the transition to 2DR regimens in other populations and contexts including low- and middle-income settings.

## Introduction

The introduction of combination antiretroviral therapy (ART) for the management of HIV has resulted in considerable improvements in survival among HIV-infected individuals and over time, regimens have become more efficacious and simpler with the need for less pills, less times a day [1–3]. Management of HIV has been based on a combination of drugs which typically include two nucleoside reverse transcriptase inhibitors (NRTI) as a "backbone" along with one non-nucleoside reverse transcriptase inhibitor (NNRTI), protease inhibitor (PI) or integrase inhibitors (also known as integrase nuclear strand transfer inhibitors or INSTIs) [4]. NRTIs have historically been associated with both short- and long-term toxicity [5] and this has led to an interest in evaluating regimens with fewer ARTs.

The integrase inhibitor drug dolutegravir (DTG) has been identified as having a clinical profile that could be suitable for an NRTI sparing, first-line two-drug regimen for the treatment of HIV. DTG has a low risk of drug-drug interaction and has been shown to be safe and effective in both treatment naïve and treatment experienced patients and regardless of baseline viral load [6–8]. DTG is also a cost-effective option [9]. DTG-based two drug regimen ART (2DR) has the potential to safely and effectively reduce patient toxicity and health care costs and increase future HIV treatment regimen options.

Two Phase III randomized clinical trials, SWORD-1 and SWORD-2, were conducted to evaluate the efficacy, safety, and tolerability of switching virologically suppressed patients from a three drug (3DR) or four drug (4DR) regimen to a 2DR regimen of DTG plus rilpivirine [10,11]. Results show that 2DR is as effective as 3DR or 4DR as maintenance therapy in patients who have already achieved viral suppression [12]. The DTG-based 2DR achieved non-inferior viral suppression (HIV-1 RNA <50 copies/milliliter) at 48 weeks compared with 3DR or 4DR and the overall rate of serious adverse events was comparable between treatment groups [12]. Similarly, GEMINI-1 and GEMINI-2 were two identical randomized clinical trials evaluating DTG plus lamivudine to a 3DR of DTG plus tenofovir disoproxil fumarate and emtricitabine. Results showed that 90% and 93% of the participants receiving the 2DR achieved plasma HIV-1 RNA <50 copies/milliliter, in the GEMINI-1 and GEMINI 2 studies respectively [13].

While 2DR may offer PLHIV who are virally suppressed, an attractive option to switch to a regimen that does not include an NRTI [14], limited information exists on patient and provider experiences with 2DR. Working in settings where the ongoing use of 2DR exists in routine clinical care, we explored perceptions of and experiences with 2DR among both patients and providers. To our knowledge, no qualitative research has been undertaken regarding these 2DR dynamics to date which can help inform further rollout.

## Materials and methods

This cross-sectional, exploratory qualitative study focused on understanding provider and patient perspectives and experiences related to 2DR. We conducted semi-structured, in-depth interviews (IDIs), among PLHIV and clinical care providers in the United States (U.S.) and Spain. Selection of the two countries was based on geographic diversity and the number of people currently using 2DR in those settings, as well as on the ground partnerships with HIV clinical care sites to facilitate patient recruitment.

We purposively interviewed 39 patients currently on DTG 2DR [dolutegravir + rilpivirine or dolutegravir + lamivudine (3TC)] and 8 clinical care providers with experience with 2DR in the U.S. and Spain. Ten to fifteen is generally considered a sufficient sample size to begin to describe a phenomenon of interest from the view of a given population group within the field of qualitative research [15]. We aimed to interview at least ten to fifteen individuals in each country to achieve saturation for that setting and to explore differences between the two countries. The patient study population included adults (≥18 years of age). We sought to include participants that providers felt would provide rich qualitative data in terms of their openness to share experiences and where possible a balance of men and women and diversity in terms of other socio-demographic factors such as age, sexual orientation, race/ethnicity at each study site. For HIV care providers, there were only a few at each clinical site where we conducted interviews and they served as key informants. Some providers and participants had experience with the Phase III SWORD-1 and SWORD-2 trials but previous trial experience or participation was not a criterion for inclusion in this study and while experience with research was an interview topic, the SWORD trials per se were not.

Clinical care providers of HIV patients on 2DR were contacted by study staff from participating study sites via phone or, in person, to arrange appointments. Study staff described study objectives and clinical care providers referred interested patients to the study investigators and qualitative interviewers. All participants provided written informed consent and interviews were conducted on site. All interactions with patient participants took place in a private room at the clinic where the patient received their ongoing HIV care and 2DR. Provider interviews also took place at the clinical site where they prescribe 2DR. Once consented, each participant was interviewed once utilizing a semi-structured IDI guide. The guides included a series of open-ended questions intended to assist in eliciting participant views, experiences and stories. Significant interviewer probing was employed.

Topics explored in the provider and patient interviews included: (1) experiences with side effects; (2) perceptions of 2DR vs. 3DR or 4DR; (3) patient satisfaction; (4) appropriate candidates for 2DR; (5) perceived clinical performance; (6) necessary support systems; and (7) future desire to continue or prescribe 2DR. All interviews were conducted by study staff trained in qualitative research techniques and research ethics. Interviews were conducted in the relevant local language, audiotaped and transcribed verbatim in their entirety for data analysis purposes.

An iterative thematic content analysis approach was utilized to approach the textual data [16]. An initial code book was developed on the basis of multiple readings of each transcript. As coding continued, additional domains of interest were documented and emerging codes were established [17]. A hierarchical coding structure was established to group codes and identify salient themes. Coding of interview transcripts was conducted using the software Atlas.ti **©** [18]. We synthesized code output per each topical area of interest and integrated that output with memos developed by each of two coders working with the data. Any discrepancies in the coding were discussed by the study team and resolved through consensus. We examined and documented shared and distinct views and experiences across the sampling categories (provider versus patient), study sites (socio-cultural context), and socio-demographic sub-groups (e.g. gender and sexual orientation, etc.).

All textual IDI data was anonymous and contained no identifiers or personal contact information. The study was approved by the Institutional Review Boards of the Johns Hopkins Bloomberg School of Public Health (#IRB00008123) and the University Hospital of Elche in Spain (#208561). Participants in the U.S. were compensated $50 for their time and in Spain, €50.

## Results

Participants included 20 patients from Spain and 19 from the U.S. (Table 1). Most were male with 17/19 participants in the U.S. and 15/20 in Spain being men. The mean age was similar across sites; most participants were in their 50s (54 in U.S.; 55 in Spain) and participant ages ranged from in the 30s to the 70s. The sample included a mix of men who have sex with men (MSM), heterosexual men and women, and bisexual individuals in both locations. The sample was more racially/ethnically diverse in the U.S. (8/19 non-Caucasian) versus Spain (20/20 Caucasian). Eight key informants (5 in Spain, 3 in the U.S.) were interviewed, all of whom were 2DR-prescribing physicians at the clinical sites.

**Table 1:** Select demographic and behavioral characteristics of study participants (n = 47).

**Patients (n = 39)**			
**Characteristic**	**Spain (n = 20)**		**U.S. (n = 19)**
Sex	15/20 Male		17/19 Male
	5/20 Female		2/19 Female
Age (mean, range)	55, 48–77		54, 38–68
Race/ethnicity	20/20 Caucasian		12/19 Caucasian
			2/19 Latino
			2/19 African American
			1/19 Asian
			1/19 Black African
			1/19 Mixed
Sexual orientation	9/20 Heterosexual male		15/19 MSM
	5/20 MSM		2/19 Heterosexual female
	5/20 Heterosexual female		2/19 Bisexual male
	1/20 Bisexual male		
**Providers (n = 8)**			
**Characteristic**		**Spain (n = 5)**	**U.S. (n = 3)**
Sex		3/5 Female	3/3 Male
		2/5 Male	
Provider type		5/5 Physician	3/3 Physician
Prescribing regimen		4/5 DTG/rilpivirine or DTG/3TC	3/3 DTG/rilpivirine
		1/5 DTG/rilpivirine	

The majority of participating PLHIV had been living with HIV for nearly two decades and had considerable experience with prior multi-drug and multi-pill ART regimens. In Spain, participants reported having been on ART for between 6 and 30 years (mean 15.2 years), while in the U.S., patients had been on ART for between 7 and 30 years (mean 17.47 years). Most participants in both countries had switched ART regimens several times prior to their most recent transition to 2DR. Patients uniformly reported that they were virally suppressed and that they had not experienced new side effects since switching to 2DR. Patients and providers in both settings noted the central role providers played in the decision to switch to a 2DR regimen and agreed that concerns about toxicity from prior multi-drug regimens were critical in the decision to switch and in satisfaction with 2DR. In the U.S. participants also stated that insurance coverage was an important factor.

### “I put my life in his hands”: Making the decision to switch to a two-drug regimen

Essentially all patients interviewed first learned of 2DR from their provider. Patients interviewed reported placing a high degree of trust in their provider and while some patients conducted additional research on 2DR, most followed their physician’s recommendation without question.

*I place a great deal of trust in the physicians*, *so I usually just take their recommendations on face value*. *I mean*, *I don’t go back and search the internet to see if there were other things that could have been—I just don’t do that*. *I just place a lot of trust in them*, *so I don’t second guess them*. [U.S. participant, male, aged 68]

*To tell you the truth*, *I have not worried much either [about the treatment that I wanted or needed]*, *like you sort of let yourself be carried away by the trust [you have in your doctor]…It is not like I am not worried*, *but I have not worried too much*, *I've always trusted him*. [Spain participant, male, aged 52]

Physicians remarked on this as well, noting that “the vast majority … defer to me” [U.S. physician] and that their patients are “always picking up on my lead” [U.S. physician].

Patients also routinely reported feeling that they could speak openly with their physicians. This sense, that there was no topic that they could not discuss freely with their clinician, further strengthened their feelings of trust:

*I feel enabled to ask questions and have an open forum with Doctor X if there were challenges*, *so I’m pretty trusting in that sense*. [U.S. participant, male, aged 57]

*Very good [communication with my doctor]*. *I mean*, *we have*, *he knows me his whole life…always when I have needed him at once I have called and then good* … *For me it is good communication*, *I totally trust [him]*. [Spain participant, female, aged 49]

### “It made me feel safer about using”: Research engagement supports both provider and patient confidence in a new regimen

Providers had either participated in SWORD trials or had tracked research on 2DR through scientific conferences and this gave them confidence when recommending the transition to 2DR for their patients.

*We have relied heavily on the results of studies*, *on the research of clinical trials*, *because at the beginning it [2DR] seemed to be prohibited*, *it had to be three drugs*…. *then we started to open our minds to it with the first results of the studies*, *then with the congresses [conferences]*, *the meetings abroad*, *and so on*. [Spain physician]

Provider engagement in research also strengthened patient trust in providers and their recommendation to switch to 2DR.

*In doing my research*, *Dr*. *X was right in the beginning involved and has done a number of studies and he’s one of the top HIV doctors in the country…And I liked the fact that he was involved in research*. *So*, *that’s why I picked him*. [U.S. participant, male, aged 55].

### “How much is taking this cutting my life?”: Concerns about the long-term toxicity of HIV treatment

Clinicians were drawn to the concept of a regimen with fewer drugs and reduced toxicity and this was a key point that they shared with their patients when introducing the option of transitioning to 2DR. Clinicians noted that when they brought up the concept of reducing toxicity, it “is something that patients have thought about” [U.S. Physician]. Indeed, the majority of patients expressed concern about the toxicity of previous regimens and this was typically a primary consideration for switching to a 2DR regimen. Several participants explained that they worried about the long-term impact that their HIV medications might have on their health.

*It's this cloud always hovering somewhere at the back of your mind that as you are taking these medications*, *what are they doing to you internally*? [U.S. participant, female, aged 44]

*Well a lot [I would worry about long-term side effects prior to 2DR]*, *because if I had them in the short term*, *imagine in the long*. *I was much more worried*, *because there were many activities I could not do*. *I could not go running [because] my stomach was hurting; I could not go for a walk* … *a thousand things*. *If you're not well* …[Spain participant, male, aged 58]

Participants highlighted a concern that exposure to HIV medications long term might shorten their life,

*You know*, *it was like*, *more than likely I'm going to die from the effects of all the medications I've taken… I remember thinking sort of midway through that eventually all these drugs were going to kill me*. [U.S. participant, male, aged 58].

or negatively affect their later quality of life,

*And that's the whole thing*, *is like okay*, *I don't want to get tackled on the one-yard line*. *Alright*? *I want to make it into the end zone*. *If the medications are there to prolong my life*, *I want to have quality of life as well*. [U.S. participant, male, aged 58]

Given these concerns, participants welcomed the concept that the 2DR regimen was doing more for them with less,

*It seems like it's a more surgical drug than what I was on five years ago*. *It does just what it's supposed to do without harm to my system*, *without other things*, *without kidney issues and heart issues and cholesterol issues and liver issues*. [U.S. participant, male, aged 57]

*For me the benefits are that you are taking less drugs* …*I think that this is already a benefit from what I see*, *that with less drugs*, *well man*, *the liver there will filter less and the blood will suck in less of all those things [that medications] carry*, *for me that is a benefit*. [Spain participant, male, aged 52]

and that ultimately a two-drug regimen would take less of a toll on their bodies.

*I’ve been on medications half my life*, *so will I do better at 75*, *if I live to be 75*, *will I do better because I’ve been on fewer medications the last 15 years of my life*? …*It’s possible that the body has less stress to it from being on one less medication*, *so I mean*, *I think our science generally tells us to treat with as few medications as possible*. [U.S. participant, male, aged 62]

*My treatment right now is magnificent*, *I am very well*. *I am delighted with the two medications the last ones that [the doctors] have given me*. *Before I used to wake up in the morning and I felt like I was so heavy that I [couldn’t] move my body*. *However*, *now I get out of bed with an energy that I did not have before*. [Spain participant, male, aged 53]

For most participants, both the size of the pill(s) that they were taking and the ease of the regimen were welcome, tangible reminders that they were on a regimen with fewer medications.

*And it makes me feel good to take it*. *It's not depressing*. *And like taking some other pills that were very large*. [U.S. participant, female, aged 52]

*I would compare that these two are much better than the other three [pills he used to take]*. *First*, *because they are two smaller pills*, *and the three that I was taking were some pills like this big…big*, *a long pill…and these are really small*, *a small sip of water and fast*. [Spain participant, male, aged 53]

### “It's needed. It's not niche”: Identifying appropriate candidates for a 2DR regimen

Patients in both the U.S. and Spain almost universally agreed that a 2DR regimen would be appropriate for any individual living with HIV regardless of whether they were treatment naïve or had significant experience with ART. Some participants highlighted young people newly diagnosed as appropriate candidates as well.

*Yeah*. *I think that everybody—that regardless of who—what do they call it*, *naive*, *when*, *you know*, *patients haven't—had it… If it was me right now*, *you know*, *just being new*, *I would definitely just look at the two drug and not even think about anything else*. (U.S. participant, male, aged 58)

Providers largely agreed with their patients.

*There's definitely people that will not qualify for that and will always be the person that is not falling into a standard category*, *but I do think the majority of people in this office would be qualified to get a 2DR regimen*. (U.S. physician)

### “I’ve been very lucky also with insurance and my employers”: Navigating cost and access

While patients in Spain are able to access the care and medicine they need without financial concern or worry given the country’s universal health care system, U.S. participants frequently mentioned insurance and the cost of medicines as a factor in their decision to switch to 2DR or in their ability to maintain a 2DR regimen. Participants remarked on the ever-present worry of continued coverage and the burden of having to manage employment as arbiter of secure access to preferred medication.

*Because what happened I just lost my coverage and there we have some problem and some miscommunication*. *So*, *I got a little scared that where would I get [it]*? [U.S. participant, male, aged 58]

In several instances, providers were actively involved in managing challenges with insurance that might impact access to 2DR.

*There was a time I was in between insurances*, *changing insurance*, *and there was a time I was in between jobs*. *And I was concerned about getting medication*, *and I was running out of supply*. *I just called my primary provider and they have—I discovered there is some way they can apply for you for emergency medication*. *And I'm telling you*, *within 24 hours*, *I already had my supply*. *I never had to worry about that again*. *…Yeah*, *that was quite cool*. [U.S. participant, female, aged 44]

## Discussion

PLHIV and their providers who participated in this qualitative research study focused on understanding perspectives and experiences related to 2DR were overwhelming positive about and satisfied with a 2DR regimen. Both patients and providers reported few side effects, noting that none had been observed since the transition to 2DR. More importantly, patients and providers both reported that 2DR was highly effective in managing HIV. In considering reasons for making the switch to 2DR, key themes that emerged from patient interviews included provider recommendation and guidance, concerns about toxicity of 3DR or 4DR regimens and for patients in the U.S. the importance of insurance to ensure access. These themes were highly salient in provider interviews as well.

Providers served as the primary gateway to 2DR for participants in both settings. Participants emphasized the trust that they placed in their providers and noted that, almost uniformly, their provider’s recommendation to switch to 2DR was the only information a patient needed to make a decision about treatment. In many instances, patients had a longstanding relationship with their provider and this was key to their trust. Many had traveled the long journey from highly disruptive regimens and worries about survival to well tolerated and highly manageable regimens such as 2DR with their providers at their side. For a majority, the switch to 2DR was a relatively simple transition in a long history of following their provider’s lead. This study builds on previous research which shows that trust in a provider clearly facilitates access to care for patients with HIV or at high risk of HIV [19–21] and suggests that regimen transitions are also facilitated by a trusting relationship between patient and provider.

Providers valued the reduced toxicity offered by 2DR and it was typically this benefit that they stressed to patients when introducing 2DR. This benefit strongly resonated with patients who had themselves been concerned about the long-term implications of being on ART. Many expressed a fear that it might be the toxicity of ART regimens and not HIV which ultimately shortened their lives. Patients and their providers had a great sense of relief about a less toxic regimen with demonstrated efficacy. Patients also had a visceral response to the smaller size of 2DR pills and the ease of the 2DR regimen and this underscored their feeling that they were on a less toxic regimen and that their HIV disease was manageable and under control. Previous research has highlighted the importance of pill size in accepting new medications and adhering to them [22–24].

In interviews with patients and providers in the U.S., insurance coverage was a constant theme. Patients noted gratitude for ‘good insurance’ and worries about maintaining coverage and losing access to 2DR if coverage was threatened. Providers were very aware of these concerns and were often engaged with their patients in ensuring continued access to medication. U.S. patients explained that their feelings of having access to the care they wanted and needed was inextricably linked to insurance coverage. Indeed, patients and providers uniformly delayed the switch to 2DR if it wasn’t covered by insurance. A recent literature review highlights the significant positive association between health insurance and higher socioeconomic status and HIV outcomes including ART adherence and virologic and immunologic response [25] underscoring the validity of the concerns raised by the U.S. participants in our research who were generally able to access treatment.

The study has several limitations including the cross-sectional nature of the research which limits our understanding of 2DR perspectives and experiences over time. Patients participating in this research were generally older individuals, who were predominantly male, many of whom had been on ART for a long period of time. A sample with greater diversity in terms of gender, age and setting might reveal differences in experiences with and perceptions of 2DR. Some of our participants had participated in clinical trials including the SWORD-1 and SWORD-2 trials, which may have given them additional insights and unique experiences. Participants’ experiences with previous studies can lead to social desirability bias. In these interviews, however, individuals spoke with interviewers that were not affiliated with either their doctor or clinic or previous research on 2DR. Further, interviewers probed interviewees about their experiences and potential concerns with a 2DR regimen at length encouraging full and detailed responses. Despite these limitations, this study has important strengths. Participants in this research reflect a depth of experience with ART that offers unique and valuable insights into the decision to transition to a 2DR regimen and experiences with that transition from both a patient and provider perspective.

This study fills a gap in the literature about the thought process through which individuals move when deciding to transition from a 3DR or 4DR regimen to a 2DR regimen. Growing evidence of the utility of DTG based 2DR regimens in clinical practice adds to the value of this research which offers foundational insights for clinicians and their treatment-experienced patients contemplating a transition to 2DR [26]. For our participants and their providers the transition to a 2DR regimen was a uniformly positive experience with essentially no reported side effects and continued viral suppression. The relationship between patients and their providers, the physical and psychological benefits of a regimen with reduced toxicity and an underlying concern about assured access through continued insurance coverage, for U.S. participants, were predominant themes in the process of contemplating and executing the switch to 2DR. This study suggests the value of conducting similar assessments among larger and more representative samples of individuals on a 2DR regimen. At the same time, results of this study will inform broader roll out of 2DR in the U.S. and Spain and form a foundation for further research on the transition to 2DR regimens in other contexts including low and middle income settings where DTG-based regimens, which are considered to be safer, more cost-effective and which have demonstrated a better barrier to the development of drug resistance, may offer a welcome alternative to traditional regimens [27].

## Supporting information

S1 Data (ZIP)
