# Supplementary material for: “It made me more confident that I have it under control”: Patient and provider perspectives on moving to a two-drug ART regimen in the United States and Spain
Source: PLoS One. 2020 May 1;15(5):e0232473. doi: 10.1371/journal.pone.0232473 (PMC7194407; doi:10.1371/journal.pone.0232473)
Supplement: S1 Data — (ZIP) [file pone.0232473.s001.zip › 2DR interview guides/2DR Guide_Provider_ENG.pdf]

**In-depth Interview guide**
**Dual regimen (2DR) – Providers**

**Interviewer**: Collect demographic data on the
Participant Information Sheet before beginning interview.

*Thank you for taking the time to speak with me today.*
*I am interested in hearing your perspectives and experiences as a clinical provider of patients on dual regimen HIV treatment.*
*I'd like to start by talking a bit about how you first became aware of the 2DR option…*

**Initial awareness & perceptions of 2DR**

- How did you first become aware of dual regimen (2DR) as an HIV treatment option?
    - Probe: When did you first become aware?
    - Probe: Who shared this information with you?
    - Probe: Where is this person based/located?

- From where else did you receive information regarding 2DR?
    - Probe: Conferences, professional groups, internet, etc.

- Tell me your initial thoughts about 2DR?
    - Probe: What concerns did you have?
    - Probe: What did you see as the benefits?
    - Probe: What did you see as the barriers?
    - Probe: What are some things you thought of with 3DR treatment that could be changed with the introduction of 2DR?

- When you think of your practice/clinical setting and the patients you see, do you think 2DR is appropriate/too niche/very needed for your population? Why?
    - Probe: What other considerations went through your mind in terms of whether 2DR might make sense for your patients?

- What and who would you say have been the biggest influencers on your thinking and decision to prescribe 2DR to your patients?
    - Probe: KOLs, local advocates, evidence based literature

**Decision to switch to 2DR**

- How many patients do you currently have on 2DR?

- How did those patients come to be on 2DR?

- Tell me about the initial conversations you had with those patients?
    - Probe: Who initiated those conversations?
    - Probe: What did you discuss?

- What made you feel like they were good candidates for 2DR?

- What specific challenges led to the switch to 2DR for these patients?
    - Probe: What would you say were the most important reasons for switching?
    - Probe: Did the most important reasons vary between you and the patient? If yes, how so?

**Candidates and criteria for 2DR**

**In-depth Interview guide**
**Dual regimen (2DR) – Providers**

- What would be an appropriate patient for a 2DR switch?
    - Probe: Are there specific 3 drug regimens where 2DR is more appropriate?

- What other patient profile or specific demographic or clinical characteristics need to be considered in a potential switch to 2DR?
    - Probe: gender, age, other behaviours (substance use, etc.)

- What are the different points in the treatment pathway where you see value for 2DR?
    - Probe: If the patient is treatment naïve, very adherent, non-adherent, has had treatment failure?

- What are your biggest concerns or worries about 2DR?
    - Probe: Any concerns about effectiveness of dual vs. triple therapy?
    - Probe: How would you say that 2DR performance compares?

- Are there specific therapies you will or will not try as part of 2DR?
    - Probe: Tell me more about this…

- What would make you stop or pull a patient off 2DR?
    - Probe: Beyond side effects, what else?

- How do you plan on introducing 2DR to your patients in the future?
    - Probe: What do you think could help other people living with HIV make a smooth transition to 2DR?

**Experience switching patients to 2DR**

- Tell me about the first patient you had on 2DR…
    - Probe: Describe the decision-making process for me…
    - Probe: Did you speak to anyone else (e.g. other clinicians, KOLs you know) who have made this switch before?

- Tell me about the most recent patient you placed on 2DR…
    - Probe: Describe the decision-making process for me…how was that experience different from the first patient you placed on this regimen?

- What types of changes have you observed that patients experience with the switch?
    - Probe: Initial reactions from your patients?
    - Probe: What are the most common side effects?
    - Probe: What about later on, do the side effects increase or decrease?

- What types of concerns have patients had?
    - Probe: How did you address those concerns?

- Tell me about any other negative experiences – treatment failure, patient discontinuation or complaint etc.
    - Probe: How did you manage those issues?

- What types of benefits do you observe that patients experience?
    - Probe: Reduced side effects

- What other types of benefits have you observed patients experience?

    - Probe: psychological, emotional benefits? Changes in how they feel about or see themselves and their HIV status related to being on dual regimen therapy?

- How have your views or perceptions of 2DR changed since you started prescribing it?
    - Probe: Do you feel more positively or negatively about it now than when you started? Why?
    - Probe: Did it meet your expectations? Fall short? Exceed?
    - Probe: Describe what you initially thought of 2DR compared to what your experience of prescribing 2DR has been?
    - Probe: Were you involved in any ViiV clinical trials prior to beginning to prescribe 2DR? How do you think your views changed from your participation in the trial?

- What types of support and communication has been important for patients during the switch?
    - Probe: What kinds of questions have they brought to you?
    - Probe: What kinds of support have they expressed they needed?

- Overall, how would you describe your experience with 2DR?

- What else would you want to share about your experience switching to 2DR?

*Thank you for your time and insights. We really appreciate this important information.*
