# Supplementary material for: “It made me more confident that I have it under control”: Patient and provider perspectives on moving to a two-drug ART regimen in the United States and Spain
Source: PLoS One. 2020 May 1;15(5):e0232473. doi: 10.1371/journal.pone.0232473 (PMC7194407; doi:10.1371/journal.pone.0232473)
Supplement: S1 Data — (ZIP) [file pone.0232473.s001.zip › 2DR interview guides/2DR Guide_Patient_SPA.pdf]

Guía para la Entrevista Detallada
Tratamiento con 2 fármacos (2DR) – Pacientes

Entrevistador: Obtenga los datos demográficos utilizando la Hoja de Información del Participante antes de comenzar la entrevista

Gracias por dedicarme su tiempo para hablar conmigo hoy. Le recuerdo que nuestra conversación es confidencial y que no hay respuestas correctas ni incorrectas. Me gustaría conocer sus opiniones y experiencias sobre su régimen actual de tratamiento para el VIH.

Me gustaría empezar preguntándole un poco acerca de su experiencia con el tratamiento para el VIH que usted esta utilizando...

Conocimiento y comprensión del tratamiento

1. ¿Puede describirme su régimen actual para el tratamiento del VIH?
    a. Indagar: ¿Cuántas pastillas toma?
    b. Indagar: ¿Cuántos fármacos (tipo de medicinas) están incluidos en su régimen?

2. ¿Puede decirme cuáles son los nombres de cada uno de los fármacos en su régimen de tratamiento?
    a. Indagar: ¿Sabe lo que hacen los fármacos? ¿Sabe cuál es el papel (objetivo) del medicamento en el tratamiento para el VIH?

3. ¿Qué otra información sabe sobre los fármacos en su régimen de tratamiento?
    a. Indagar: ¿Qué efectos secundarios puede causar su tratamiento para el VIH?
    b. Indagar: ¿Qué instrucciones (indicaciones) tiene su tratamiento para el VIH de cómo y cuándo puede tomar los medicamentes?

Experiencias previas con el tratamiento para el VIH

4. Hábleme más específicamente sobre el régimen que estaba tomando antes de cambiar a su régimen actual...
    a. Indagar: ¿Cuáles eran los nombres de los fármacos para el VIH que estaba tomando antes del cambio?
    b. Indagar: ¿Cuánto tiempo estuvo tomando ese régimen?
    c. Indagar: Hábleme más sobre su experiencia con ese régimen en particular ...

5. En general ¿durante cuánto tiempo ha estado tomando TAR?
    a. Indagar: ¿Cuántas veces ha cambiado de tratamiento incluyendo su actual tratamiento?
    b. Indagar: ¿Describiría su experiencia con el TAR antes de su tratamiento actual como positiva o negativa? ¿Por qué?

6. Qué tipo de efectos secundarios ha tenido en el pasado antes de empezar su régimen actual?
    a. Indagar: ¿Cómo manejó los efectos secundarios?
    b. Indagar: ¿Qué otros problemas o preocupaciones tuvo con sus fármacos para el VIH antes de empezar su régimen actual?
    c. Indagar: ¿Cómo cambiaron estas experiencias o preocupaciones a lo largo del tiempo?

7. ¿Qué tan preocupado estaba por los efectos a largo plazo de su anterior tratamiento?
    a. Indagar: Hábleme más sobre estas preocupaciones…
    b. Indagar: ¿Cómo manejó estas preocupaciones?
    c. Indagar: ¿Habló sobre estas preocupaciones con alguien?

Control y conocimiento del tratamiento

8. ¿Qué tipo de conversaciones ha tenido con su(s) médico(s) sobre el tratamiento para el VIH a lo largo de los años?
    a. Indagar: ¿De qué tipo de cosas hablaron?
    b. Indagar: ¿Qué le parece la comunicación que tiene con su(s) médico(s) sobre el tratamiento para el VIH?
    c. Indagar: ¿Hay cosas de las que le gustaría poder hablar más abiertamente?

9. ¿Hasta qué punto cree que ha tenido control en el pasado sobre su tratamiento para el VIH?
    a. ¿Hasta qué punto cree que ha estado bien informado acerca de sus opciones de tratamiento?
    b. ¿Cree que pudo obtener el tratamiento que quería/necesitaba?

10. Cuando piensa en sus experiencias tomando TAR, ¿qué diría que es lo más importante para usted sobre el tratamiento para el VIH?
    a. Indagar: ¿La facilidad/conveniencia (menos pastillas, pastillas más pequeñas)? ¿Los efectos secundarios (cantidad o gravedad)? ¿La eficacia?
    b. Indagar: ¿Qué más es lo más importante para usted sobre un tratamiento para el VIH?

11. Pensando en sus propias experiencias tomando TAR, ¿cuáles son algunas de las razones por las que usted cree que una persona puede tomar un medicamento versus otro o cambiar de un régimen de tratamiento a otro?

Conocimiento inicial del régimen de dos fármacos (2DR)

12. ¿Cómo se enteró por primera vez sobre el régimen de dos fármacos (2DR), el cual usted está tomando actualmente como tratamiento para el VIH?
    a. Indagar: ¿Cuándo se enteró de este régimen por primera vez?
    b. Indagar: ¿Quién compartió esa información con usted?
    c. Indagar: ¿Dónde está ubicada (o se encuentra) esa persona?

13. ¿Hábleme más sobre sus primeras conversaciones sobre el régimen de dos fármacos (2DR)?
    a. Indagar: ¿Qué conversaciones tuvo con sus médicos?
    b. Indagar: ¿ Qué conversaciones tuvo con familiares y amigos?
    c. Indagar: ¿Con quién más habló sobre 2DR?

14. ¿Qué fue lo que usted entendió (comprendió) sobre la diferencia entre este régimen (2DR) y el régimen anterior que usted estaba tomando?

15. ¿Qué otra información recibió con respecto al régimen de dos fármacos (2DR)?
    a. Indagar: ¿Algún material que le dio su médico? ¿Que era?
    b. Indagar: Alguna información encontrada en el Internet, chats en línea, grupos de apoyo, etc. ¿Qué incluye esa información?

Decisión de cambiar al régimen de dos fármacos (2DR)

16. ¿Por qué se interesó en aprender más sobre el régimen de dos fármacos (2DR)?

17. ¿Qué específicamente le hizo querer cambiar al régimen de dos fármacos (2DR)?
    a. Indagar: Si pudiera nombrar una cosa que le hizo querer cambiar, ¿cuál sería?

18. ¿Hábleme más sobre el proceso de cómo tomó la decisión de cambiar al régimen de dos fármacos (2DR)?
    a. Indagar: ¿Con quién habló, qué factores consideró, etc.?

19. ¿Qué preocupaciones o miedos tenía relacionados al cambio de régimen de fármacos?
    a. Indagar: ¿Alguien más expresó preocupación acerca de que usted haya cambiado de régimen de fármacos?

Opiniones y experiencias con el régimen de dos fármacos (2DR)

20. Hábleme de su experiencia con el régimen de dos fármacos (2DR), el cual usted esta tomando actualmente…
    a. Indagar: ¿Durante cuánto tiempo ha estado tomando el régimen de dos fármacos (2DR)?

21. ¿Qué cambios ha experimentado desde el cambio?
    a. Indagar: ¿Qué tipo de efectos secundarios ha tenido?
    b. Indagar: ¿Qué ha hecho para controlar esos efectos secundarios?
    c. Indagar: ¿Han aumentado o disminuido con el tiempo?

22. ¿Cuáles han sido los beneficios más importantes que ha experimentado desde el cambio?
    a. Indagar: ¿Menor toxicidad, menos efectos secundarios?

23. ¿Qué otros beneficios observa con el régimen de dos fármacos (2DR)?
    a. Indagar: ¿Ve algún beneficio psicológico? ¿Ha cambiado su actitud o estado de ánimo? Hábleme más sobre esto…
    b. Indagar: ¿Hay otras comodidades asociadas con el régimen de dos fármacos (2DR)?

24. ¿Cuáles han sido sus mayores preocupaciones con el régimen de dos fármacos (2DR)?
    a. Indagar: ¿Tuvo alguna preocupación acerca de la eficacia del régimen de dos fármacos (2DR) en comparación con el tratamiento de tres fármacos?
    b. Indagar: ¿Cómo se compara el régimen de dos fármacos con su tratamiento anterior?

25. Ahora pensando en términos globales, ¿cuáles considera que son los beneficios y desventajas de tener 2 fármacos en lugar de 3 o 4 fármacos en su régimen?
    a. Indagar: ¿Cuáles cree que son los beneficios?
    b. Indagar: ¿Hay algún beneficio emocional o psicológico relacionado con tomar un régimen de 2 fármacos versus uno de 3 o 4 fármacos?
    c. Indagar: ¿Un régimen de tratamiento afecta cómo usted se siente consigo mismo o cómo se ve a si mismo más que otro régimen de tratamiento? De qué manera….
    d. Indagar: ¿Es un régimen de tratamiento más o menos estigmatizado que el otro? De qué manera….
    e. Indagar: ¿Cuáles crees que son las desventajas?

26. ¿Cómo han cambiado sus puntos de vista o sus percepciones sobre el régimen de dos fármacos (2DR) desde que comenzó a tomarlo?
    a. Indagar: ¿Te gusta más ahora que cuando empezaste a tomarlo? ¿Te parece peor ahora? ¿Por qué?
    b. Indagar: ¿Cumplió con sus expectativas? ¿No cumplió con sus expectativas (hubieron faltas)? ¿Excedió (fue más haya) de sus expectativas? ¿De qué manera?
    c. Indagar: Describa lo que pensó sería la experiencia de estar en el régimen de dos fármacos (2DR) en comparación con lo que ha sido su experiencia (real) estando con el régimen de dos fármacos (2DR).
    d. Indagar: ¿Estuvo involucrado en algún estudio clínico de ViiV antes de comenzar con el régimen de dos fármacos (2DR)? ¿Cómo cree que cambiaron sus puntos de vista o percepciones por su participación en el estudio clínico?

27. En general, ¿hasta qué punto está satisfecho con el régimen de dos fármacos (2DR)?
    a. Indagar: ¿Le gustaría continuar con el régimen de dos fármacos (2DR)?
    b. Indagar: ¿Qué le haría dejar de querer tomarlo?

28. ¿Recomendaría el régimen de dos fármacos (2DR) a otras personas que viven con el VIH?
    a. Indagar: ¿Cree que hay tipos concretos de personas que piensa que serían mejores candidatos para el régimen de dos fármacos (2DR)?
    b. Indagar: ¿Qué cree que podría ayudar a otras personas con VIH a realizar una transición suave al régimen de dos fármacos (2DR)?

29. ¿Qué más le gustaría compartir sobre su experiencia con el cambio al régimen de dos fármacos (2DR)?

Gracias por su tiempo y sus opiniones. Apreciamos mucho esta información importante.
