# Supplementary material for: “It made me more confident that I have it under control”: Patient and provider perspectives on moving to a two-drug ART regimen in the United States and Spain
Source: PLoS One. 2020 May 1;15(5):e0232473. doi: 10.1371/journal.pone.0232473 (PMC7194407; doi:10.1371/journal.pone.0232473)
Supplement: S1 Data — (ZIP) [file pone.0232473.s001.zip › 2DR interview guides/2DR Guide_Patient_ENG.pdf]

In-depth Interview Guide
Dual regimen (2DR) – Patients

Interviewer: Collect demographic data on the Participant Information Sheet before beginning interview.

Thank you for taking the time to speak with me today. As a reminder, our conversation is confidential and there are no right or wrong answers. I am interested in hearing about your views and experiences with your current HIV treatment regimen.
I'd like to start by asking you to tell me a little bit about the HIV treatment you are on…

Treatment knowledge and literacy

- Can you describe your current HIV treatment regimen to me?
    - Probe: How many pills do you take?
    - Probe: How many drugs does your regimen include?

- Can you tell me what the names are of each of the drugs in your treatment regimen?
    - Probe: Do you know what the drugs do? Do you know what their role is in treating HIV?

- What other information do you know about the drugs in your treatment regimen?
    - Probe: What side effects do you know of that your HIV treatment may cause?
    - Probe: What instructions does your HIV treatment have for when or how to take it?

Prior HIV treatment experiences

- Tell me about the specific regimen you were on before switching to your current regimen…
    - Probe: What were the names of the drugs you were on for HIV prior to the switch?
    - Probe: How long had you been on that regimen?
    - Probe: Tell me more about your experience with that particular regimen…

- How long have you been taking ART overall?
    - Probe: How many times have you switched regimens including this most recent switch?
    - Probe: Would you describe your experience taking ART prior to your current regimen as positive or negative? Why?

- What types of side effects did you have in the past prior to starting your current regimen?
    - Probe: How did you manage those side effects?
    - Probe: What other challenges or concerns did you have about your HIV medications prior to being on your current regimen?
    - Probe: How did those experiences or concerns change over time?

- How worried were you about the long-term effects of your prior regimen?
    - Probe: Tell me more about these concerns…
    - Probe: How did you manage these concerns?
    - Probe: Did you discuss these concerns with anyone?

In-depth Interview Guide
Dual regimen (2DR) – Patients

Treatment agency

- What types of conversations have you had with your clinician(s) about your HIV treatment over the years?
    - o Probe: What types of things did you discuss?
    - o Probe: How do you feel about the communication you have had with your provider(s) about your HIV treatment?
    - o Probe: Are there things you wish you could talk more openly about?

- How much control do you feel you have had in the past over your HIV treatment?
    - o How well informed do you feel you were about what your treatment options were?
    - o Do you feel like you were able to get the treatment you wanted/needed?

- When you think about your experiences taking ART, what would you say is the single most important thing for you about an HIV treatment?
    - o Probe: Ease/convenience (less pills, smaller pills), side effects (amount or severity), efficacy?
    - o Probe: What else is most important to you about an HIV treatment?

- When you think about your own experiences taking ART, what are some reasons you can think of why a person might take one medication versus another or switch from one treatment regimen to another?

Initial awareness of 2DR

- How did you first find out about the dual regimen (2DR) you are currently on as an HIV treatment option?
    - o Probe: When did you first become aware?
    - o Probe: Who shared this information with you?
    - o Probe: Where is this person based/located?

- Tell me about your initial conversations you had regarding 2DR?
    - o Probe: What conversations did you have with your clinicians?
    - o Probe: What about with family and friends?
    - o Probe: Who else did you discuss 2DR with?

- What did you understand to be the difference between this regimen compared to what you had been on previously?

- What other information did you receive regarding 2DR?
    - o Probe: Materials from your provider? What were they?
    - o Probe: Information from the internet, online chats, support groups, etc.? What did that include?

Decision to switch to 2DR

- What made you interested in learning more about 2DR?

In-depth Interview Guide
Dual regimen (2DR) – Patients

- What specifically made you want to make the switch to 2DR?
  - Probe: If you could name one thing that made you want to switch, what would it be?

- Tell me more about the decision-making process itself regarding the switch to 2DR?
  - Probe: Who did you speak to, what factors did you consider, etc.?

- What concerns or fears did you have about switching drug regimens?
  - Probe: Did anyone else express concern about you switching regimens?

Views and experiences with 2DR

- Tell me about your experience with the 2DR treatment you are on now…
  - Probe: How long have you been on 2DR?

- What changes have you experienced since the switch?
  - Probe: What types of side effects have you had?
  - Probe: What have you done to manage those side effects?
  - Probe: Have those side effects increased or decreased with time?

- What have been the most important benefits that you have experienced since switching?
  - Probe: Less toxicity, lower side effects?

- What other benefits do you see with your current 2DR?
  - Probe: Do you see any psychological benefits? Has it changed your outlook or mood at all? Tell me more about that…
  - Probe: Are there any other conveniences associated with 2DR?

- What have been your biggest concerns with 2DR?
  - Probe: Any concerns about effectiveness of dual vs. triple therapy?
  - Probe: How would you say that the 2DR performance compares to your prior regimen?

- Thinking broadly now, what do you see as the benefits and drawbacks of having 2 drugs rather than 3 or 4 drugs in your regimen?
  - Probe: What do you think are the benefits?
  - Probe: Are there any emotional or psychological benefits to being on a 2-drug versus a 3- or 4-drug regimen?
  - Probe: Does one affect how you feel about yourself or see yourself more than the other? In what way….
  - Probe: Is one more or less stigmatizing than the other? In what way….
  - Probe: What do you think are the drawbacks?

- How have your views or perceptions of 2DR changed since you started taking it?
  - Probe: Do you like it better now than when you started? Worse? Why?
  - Probe: Did it meet your expectations? Fall short? Exceed? In what ways?

    - Probe: Describe what you thought would be the experience of being on 2DR compared to what your experience of being on 2DR has been?
    - Probe: Were you involved in any ViiV clinical trials prior to starting on 2DR? How do you think your views changed from your participation in the trial?

- Overall, how satisfied are you with 2DR?
    - Probe: Would you like to continue with 2DR?
    - Probe: What would make you want to stop?

- Would you recommend 2DR to other people living with HIV?
    - Probe: Are there any specific types of people you think would be better candidates for 2DR?
    - Probe: What do you think could help other people living with HIV make a smooth transition to 2DR?

- What else would you want to share about your experience switching to 2DR?

Thank you for your time and insights. We really appreciate this important information.
