# Supplementary material for: “It made me more confident that I have it under control”: Patient and provider perspectives on moving to a two-drug ART regimen in the United States and Spain
Source: PLoS One. 2020 May 1;15(5):e0232473. doi: 10.1371/journal.pone.0232473 (PMC7194407; doi:10.1371/journal.pone.0232473)
Supplement: S1 Data — (ZIP) [file pone.0232473.s001.zip › 2DR interview guides/2DR Guide_Provider_SPA.pdf]

Guía para la Entrevista Detallada
Tratamiento con 2 fármacos (2DR) – Profesionales sanitarios

Entrevistador: Obtenga los datos demográficos utilizando la Hoja de Información del Participante antes de comenzar la entrevista.

Gracias por dedicarme su tiempo para hablar conmigo hoy.
Estoy interesado en escuchar sus perspectivas y experiencias como profesional sanitario que trata a pacientes que están recibiendo tratamiento dual para el VIH.

Me gustaría empezar hablando un poco sobre cómo conoció la opción del régimen de dos fármacos (2DR)…

Conocimiento y percepciones iniciales del 2DR

- ¿Cómo conoció la existencia del tratamiento con 2DR (por sus siglas en inglés, 2 *drug regime*) como una opción de tratamiento para el VIH?
    - Indagar: ¿Cuándo supo de su existencia?
    - Indagar: ¿Quién compartió esta información con usted?
    - Indagar: ¿Dónde reside/se encuentra esta persona?

- ¿De qué otras fuentes recibió información sobre los 2DR?
    - Indagar: Conferencias, grupos profesionales, internet, etc.

- Hábleme sobre su opinión inicial del 2DR
    - Indagar: ¿Qué le preocupaba?
    - Indagar: ¿Qué es lo que considera como los beneficios?
    - Indagar: ¿Qué es lo que considera como las barreras?
    - Indagar: ¿Cuáles son algunas de las cosas que pensaba del tratamiento triple (TT) que podrían modificarse con la introducción del 2DR?

- Cuando piensa en su centro médico/consulta y en los pacientes a los que atiende, ¿cree que el 2DR es apropiado/muy específico/muy necesario para su población? ¿Por qué?
    - Indagar: ¿Qué otras consideraciones le pasaron por la cabeza en términos de si el 2DR podría tener sentido para sus pacientes?

- ¿Qué y quién diría que han sido las mayores influencias en su forma de pensar y en la decisión de prescribir el 2DR a sus pacientes?
    - Indagar: Líderes de opinión, defensores locales, publicaciones basadas en evidencias

Decisión de cambiar al 2DR

- ¿Cuántos pacientes tiene actualmente que reciben 2DR?

- ¿Cómo llegaron esos pacientes a recibir el 2DR?

- Hábleme sobre las conversaciones iniciales que tuvo con esos pacientes

- Indagar: ¿Quien inició las conversaciones?
- Indagar: ¿De qué hablaron?

- ¿Qué le hizo pensar que eran buenos candidatos para el 2DR?

- ¿Qué desafíos específicos le llevaron a cambiar a estos pacientes al 2DR?
    - Indagar: ¿Cuáles diría que fueron las razones más importantes para el cambio?
    - Indagar: ¿Las razones más importantes variaron entre usted y el paciente? Si la respuesta es afirmativa, ¿de qué manera?

Candidatos y criterios para el 2DR

- ¿Quién sería un paciente apropiado para el cambio al 2DR?
    - Indagar: ¿Existen tratamientos farmacológicos triples específicos en los que el 2DR es más apropiado?

- ¿Qué otro perfil de paciente o características demográficas o clínicas específicas deben tomarse en cuenta en un posible cambio al 2DR?
    - Indagar: sexo, edad, otros comportamientos (consumo de sustancias, etc.)

- ¿Cuáles son los diferentes puntos en el curso del tratamiento donde ve valor para el 2DR?
    - Indagar: Si el paciente no ha recibido tratamiento previo, cumple estrictamente el tratamiento, no cumple el tratamiento, ha fracasado con el tratamiento

- ¿Cuáles son sus principales inquietudes o preocupaciones sobre el 2DR?
    - Indagar: ¿Tuvo alguna preocupación acerca de la eficacia del tratamiento dual en comparación con el tratamiento triple?
    - Indagar: ¿Como diría que es el rendimiento del 2DR en comparación?

- ¿Existen tratamientos específicos que podría probar o no como parte del 2DR?
    - Indagar: Hábleme más sobre esto…

- ¿Qué le haría detener o retirar a un paciente del 2DR?
    - Indagar: Además de los efectos secundarios, ¿qué más?

- ¿De qué manera piensa presentar el 2DR a sus pacientes en el futuro?
    - Indagar: ¿Qué cree que podría ayudar a otras personas con VIH a realizar una transición suave al 2DR?

Experiencia al cambiar a los pacientes al 2DR

- Hábleme sobre el primer paciente que tuvo que recibió 2DR…
    - Indagar: Descríbame el proceso de toma de la decisión…
    - Indagar: ¿Habló con alguien más (por ejemplo, otros médicos, líderes de opiniones, que conozca) que haya hecho este cambio antes?

Guía para la Entrevista Detallada
Tratamiento con 2 fármacos (2DR) – Profesionales sanitarios

- Cuénteme sobre el paciente más reciente que colocó en 2DR...
  - Indagar: Describa para mí el proceso de toma de decisiones... ¿cómo fue esa experiencia diferente a la del primer paciente que usted colocó en este régimen?

- ¿Qué tipos de cambios ha observado que hayan experimentado los pacientes con el cambio?
  - Indagar: ¿Cuáles fueron las reacciones iniciales de sus pacientes?
  - Indagar: ¿Cuáles son los efectos secundarios más frecuentes?
  - Indagar: Y más adelante, ¿los efectos secundarios aumentaron o disminuyeron?

- ¿Qué tipos de preocupaciones tenían los pacientes?
  - Indagar: ¿Cómo abordó esas preocupaciones?

- Háblame sobre cualquier otra experiencia negativa: fracaso del tratamiento, abandono o queja del paciente, etc.
  - Indagar: ¿Cómo abordó esas cuestiones?

- ¿Qué tipos de beneficios observó en los pacientes?
  - Indagar: Disminución de los efectos secundarios

- ¿Qué otros tipos de beneficios ha observado en los pacientes?
  - Indagar: ¿beneficios psicológicos, emocionales? ¿Cambios en cómo se sienten o se ven a sí mismos y su estado de VIH relacionado con el tratamiento doble?

- ¿Cómo han cambiado sus puntos de vista o percepciones sobre el 2DR desde que comenzó a recetarlo?
  - Indagar: ¿Se siente más positivo o negativo sobre la prescripción del 2DR ahora que cuando comenzó a recetarlo? ¿Por qué?
  - Indagar: ¿Cumplió con sus expectativas? ¿Hubieron faltas? ¿Excedió?
  - Indagar: Describa lo que inicialmente pensó sobre el 2DR en comparación con lo que ha sido su experiencia recetando el 2DR.
  - Indagar: ¿Estuvo involucrado en algún estudio clínico de ViiV antes de comenzar a recetar el 2DR? ¿Cómo cree que cambiaron sus puntos de vista o percepciones por su participación en el estudio?

- ¿Qué tipos de apoyo y comunicación han sido importantes para los pacientes durante el cambio?
  - Indagar: ¿Qué tipo de preguntas le han hecho?
  - Indagar: ¿Qué tipo de apoyo han expresado que necesitaban?

- En general, ¿cómo describiría su experiencia con el 2DR?

- ¿Qué más le gustaría compartir sobre su experiencia con el cambio al 2DR?

Gracias por su tiempo y sus opiniones. Apreciamos mucho esta información importante.
